# Food environment trajectories: a sequence analysis from the CARTaGENE cohort

**DOI:** 10.1017/S1368980024000119

**Published:** 2024-01-22

**Authors:** Habila Adamou, Éric Robitaille, Marie-Claude Paquette, Alexandre Lebel

**Affiliations:** 1 Center for Research in Regional Planning and Development (CRAD), Laval University, Quebec, Canada; 2 Evaluation Platform on Obesity Prevention, Quebec Heart and Lung Institute Research Center, Quebec, Canada; 3 Institut national de santé publique du Québec, 190, boulevard Crémazie Est, Montréal, Québec, Canada; 4 ESPUM, Université de Montréal, Montréal, Canada; 5 Département de nutrition, Université de Montréal, Montréal, Canada

**Keywords:** Food environment trajectory, Sequence analysis, Food stores, Food environment

## Abstract

**Objective:**

The purpose of this study was to create a typology of longitudinal exposure to food environment based on socio-economic context.

**Design:**

Food environment trajectories were modelled using a sequence analysis method, followed by a logistic regression to describe those trajectories.

**Setting:**

The study took place in Quebec, Canada, using food environment data from 2009, 2011 and 2018 merged with participants’ demographic and socio-economic characteristics.

**Participant:**

At recruitment, 38 627 participants between the ages of 40 and 69 years from six urban areas in Quebec were included in the CARTaGENE cohort study. The cohort was representative of the Quebec urban population within this age range.

**Results:**

Our study revealed five trajectories of food access over time: (1) limited access to food stores throughout the study period, (2) limited access improving, (3) good access diminishing, (4) good access throughout the period and (5) low access throughout the period. Logistic regression analysis showed that participants who were unable to work (OR = 1·42, CI = 1·08–1·86), lived in households with five or more persons (OR = 1·69, CI = 1·17–2·42) and those living in low-income households (OR = 1·32, CI = 1·03–1·71) had higher odds of experiencing a disadvantaged food environment trajectory. Additionally, the level of education and age of participants were associated with the odds of experiencing a disadvantaged food environment trajectory.

**Conclusions:**

The study demonstrates that people facing socio-economic disadvantage are more likely to experience a disadvantaged food environment trajectory over time.

Diet plays a major role in determining the health status of a population^(1)^. In 2019, the most important risk factors associated with mortality and morbidity in Canada were smoking, high BMI, high blood pressure, high fasting glucose and poor diet^(2)^. The adoption of a healthy diet depends not only on individual determinants (e.g. food preferences, nutritional knowledge and psychological factors) but also on environmental determinants such as the characteristics of the physical, economic, political and sociocultural environments (e.g. family context, physical and economic access to and availability of food, social status, and income)^(3,4)^.

The effects of the characteristics of the food environment (e.g. accessibility to food stores and food policies) on population health have received increased attention in recent years. Most studies have focused on associations between food environment characteristics and chronic disease^(5–8)^, diet quality^(9)^, the quality of the food supply^(10–13)^, and fruit and vegetable consumption^(10,13)^. Of the chronic diseases, obesity is most often used to measure the impact of the food environment on population health^(7,9,14–16)^. Other studies have explored the links between neighbourhood socio-economic status and the food environment to which individuals are exposed^(11,12,17)^. Much of this research focuses on the food environment around the schools or homes of young people^(5,18–22)^ to explore its links with childhood obesity, diet quality and food supply quality.

The food environment is complex, and developing indicators to characterise it reliably is particularly challenging^(23–25)^. Most measurements that exist can be grouped into three broad categories: availability, accessibility and quality of food offerings^(24,25)^. The most common types of food sources used to develop these measurements are grocery stores, supermarkets and fast-food restaurants^(24)^. Food availability is usually measured using the number of food sources, the density per area or the ratio of food sources to people^(11,14–16,18)^. Accessibility is measured using Euclidean or network distances between food sources and the nearby residential locations, schools, or workplaces^(10,13,17,26,27)^. Finally, studies most often measure the quality of food offered by analysing the food supply offered or by calculating a food quality index^(5,10,18)^. Food sources are often identified using an existing classification of commercial stores or lists of specific store names^(22)^.

Most of these studies of food environment report weak associations with health indicators or diet quality^(5,16,18)^. Studies showing significant associations between the food environment and weight status or eating behaviours primarily investigated urban and low socio-economic environments^(7,14)^. Most used cross-sectional designs^(7)^ and did not control for the duration of exposure to a given food environment, which could explain the weak associations.

Understanding the relationship between food environments and population health may hinge on considering both the duration and the trajectory of exposure to these environments^(28–30)^. More recent studies elaborated new strategies to measure the exposition of individuals’ food environment trajectories in time. Many studies used longitudinal analysis for identifying trajectories of access to healthy food store type^(17,31)^, unhealthy food outlets^(12,32)^ or both^(31,32)^. Other strategies aimed at understanding changes in the food environment over time by simultaneously measuring exposure to the home food environments, the workplace food environments and the food environment along home–work commutes^(26)^. The relationship between exposure and utilisation was also recognised to be influenced by the temporal and spatial context within which individuals encounter food retailers^(29)^.

Results from these studies reported various findings for several health issues such as obesity or food consumption. However, all studies concluded to some extent that individuals residing in socio-economically disadvantaged conditions showed higher exposure to long-term unhealthy food environment trajectories and had a diminished supply of health-promoting foods in comparison to more affluent communities^(33–35)^. Some further observed that the weekly consumption of fast food among individuals was linked to an unhealthy food environment and elevated fast-food restaurant density, especially within disadvantaged communities^(10,33)^, but that this disparity may dissipate over time due to larger increases in proximity to fast food in wealthier neighbourhoods^(26)^.

However, all studies acknowledged that measuring exposure to the food environment is challenging and has limitations in comprehending the intricate relationship between food store availability and healthy eating. They often have shortcomings such as the reliance on inaccurate commercial databases for food establishment data, heterogeneity of geographic measurements or indexes used, and the availability of representative longitudinal individual-level data along with precise geographic information. Furthermore, despite many studies, associations between food environments and health are often inconsistent since results vary importantly according to political and socio-economic context. These limitations prevent policymakers from a clear description to address public health challenges related to the food environment. Longitudinal approaches adapted to political context are thus needed to orient policymakers to better address issues related to the food environment under their jurisdiction.

The objective of this study was to create a typology of longitudinal exposure to urban food environments by socio-economic context in Quebec.

## Materials and methods

We utilise sequence analysis to 1) create food environment trajectories and a 2) typology of these trajectories. Additionally, logistic regression models were used to characterisze disadvantaged food environment trajectories based on demographic and socio-economic characteristics. In our study, logistic regression allowed us to estimate the likelihood of a participant being in a disadvantaged food environment trajectory according to demographic and socio-economic variables. All analyses were done in 2022.

### Individual data: sample and variables

CARTaGENE is a publicly funded research platform that was developed in 2003 to facilitate health research and support decision-making in Quebec, Canada. The platform comprises a population-based cohort of 43 000 participants from six metropolitan areas in the province. The CARTaGENE cohort is an ongoing study that includes participants from both Phase A (2009–2010) and Phase B (2013–2014) recruitment periods. The study focuses on participants aged 40–69 years, who were representative of the urban Quebec population in this age group at the time of recruitment. CARTaGENE is the largest prospective study of adult health in Quebec and includes both biological samples and individual data. The platform aims to reduce healthcare costs and promote public health by providing a valuable tool for researchers and decision-makers. Data were collected on demographics and socio-economic characteristics, physical and mental health, nutrition, and living environments. Participants’ administrative data from Quebec’s health insurance plan (Régie de l’assurance maladie du Québec (RAMQ)) was combined with the CARTaGENE data. More information on the recruitment, development and data management of the CARTaGENE cohort is available in this reference^(36)^ and on the platform’s website https://cartagene.qc.ca/. Participants’ sex, age, the highest level of education, occupational status, marital status, annual household income and the number of dependents in the household were obtained from the CARTaGENE cohort data.

### Participant selection

#### Food environment data

Data on the food environment were collected by Quebec’s public health institute (INSPQ, Institut national de santé publique du Québec). The data on the food environment in Quebec include information about the availability and proximity of food sources. The information is gathered based on the 2016 census dissemination area (DA), which is the smallest spatial unit in the Canadian census that provides socio-economic data^(37)^. Our definition of food environment is based on a food store access index created by Quebec’s public health institute (INSPQ). This index is available for 2009, 2011 and 2018, and it measures the accessibility of food stores such as grocery stores, supermarkets, farmer’s markets, and fruit and vegetable shops. In our study, the food stores access index was categorised as: 1. food desert, 2. limited access to food stores and 3. favourable access to food stores. Accessibility of food stores was calculated using an area where the centre is geographically weighted according to residential distribution and the nearest food store. One kilometre or more is used to define low access to food stores in urban areas. A food desert is defined as a DA with low access to food stores which is also in the most materially disadvantaged quintile^(38)^. For more detailed information on the food store access index, see Robitaille and Bergeron^(38)^.

**Fig. 1:**
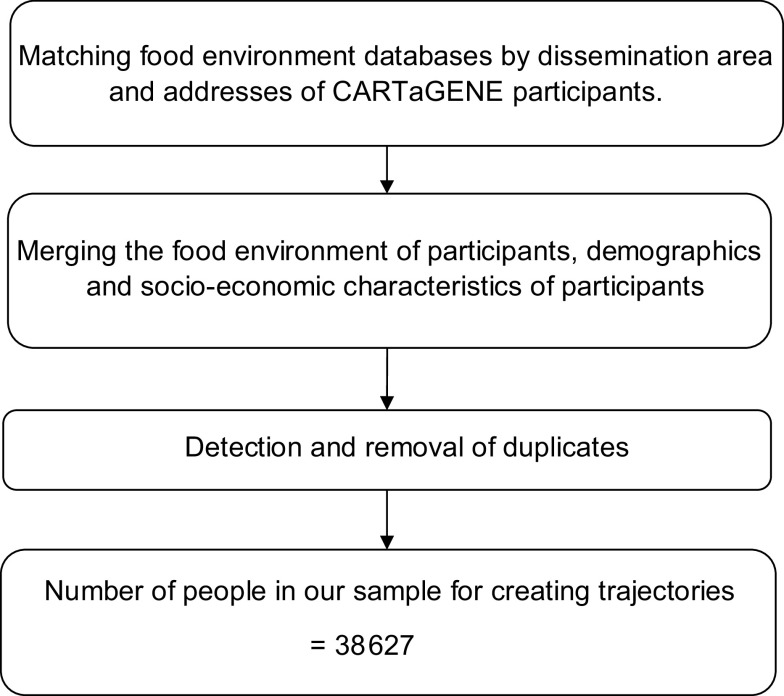
Sample selection process

Our study involved matching the food store access index with the respective DA where participants lived in 2009, 2011 and 2018. This allowed us to obtain the food store access index for each participant in the CARTaGENE cohort for those years. The procedure is shown in Fig. 1.

### Construction of food environment trajectories

To classify and differentiate imperceptible subgroups of sequences based on their reactions to a collection of detectable indicators (food access stores), we employ sequence analysis followed by optimal matching to determine the requisite conversions among the various modalities of food access stores. Ultimately, we employ inertia jumps to determine the number of classes to be chosen.

Sequence analysis is an exploratory classification methodology designed to unveil patterns in data^(39–41)^, providing a condensed representation of the sample. Sequence analysis, employed to discern sequence patterns, transitions and temporal trends, facilitates the identification of latent subgroups of individuals based on their responses to a set of observable indicators. This leads to trajectory construction. In our case, each trajectory is described by a sequence, that is, by a chronologically ordered sequence of elementary ‘access to food stores’. We use optimal matching to compare dissimilarities between sequence pairs. Then, hierarchical ascending classification to group sequences into several classes based on their proximity.

Optimal matching, a key approach, involves determining, for each pair of sequences, the minimum number of substitutions (where one element is replaced with another), deletions (where one element is removed) and insertions (where one element is added) needed to align them. In this study, optimal matching sequence analysis computed dissimilarity between sequence pairs in the sample^(39–41)^. Subsequently, a sequence typology was constructed, grouping similar sequences through hierarchical ascending classification, where costs were computed based on application-specific criteria.

Although attributing costs to social distance in the social sciences is challenging, a matrix of substitution costs was employed, where all costs were constant and set at 2 (Table 1). The calculated distance between sequences incorporated an insertion/deletion (indel) cost equal to 1^(41,42)^. The primary goal was to ascertain whether the sequence order within trajectories justifies an indel value of 1.

R (R Core Team, 2019) TraMineR package^(43)^ facilitated sequence analyses. The classification iteratively grouped individuals with similar experiences in successive food environments from 2009 to 2018. The resulting information was presented as a dendrogram – a classification tree – where each level represented a subset of individuals. This dendrogram, based on inertia jumps, aided in determining the number of classes. Hierarchical ascending classification associated with Ward’s criterion was utilised for trajectory typologies, seeking to minimise heterogeneity within classes while maximising differences between classes. This approach identified five classes of food store access trajectories for CARTaGENE cohort participants between 2009 and 2018, denoted as food environment trajectories.

## Results

### Sample characteristics

Table 2 provides information on the characteristics of the participants based on the variables included in the analyses. Many of the participants were female (56 %) and married (67 %). Additionally, 47 % of the participants held a university degree, 70 % were employed and 56 % had favourable access to food stores in 2009, 52 % in 2011 and 49 % in 2018.

**Table 1:** Matrix of insertion, suppression and substitution costs between the three modalities of the classification variable

Food access	Food desert	Limited access	Favourable access
Food desert	0	2	2
Limited access	2	0	2
Favourable access	2	2	0

**Table 2:** Distribution of participants by variable included in the analyses

Variable	Participants (%)	
*Food access 2009*		
Food desert	1492	3·86 %
Limited access to food stores	15 291	39·59 %
Favourable access to food stores	21 844	56·55 %
*Food access 2011*		
Food desert	1338	3·46 %
Limited access to food stores	17 001	44·01 %
Favourable access to food stores	20 288	52·52 %
*Food access 2018*		
Food desert	1744	4·51 %
Limited access to food stores	17 808	46·10 %
Favourable access to food stores	19 075	49·38 %
*Sex at birth*		
Female	21 635	56 %
Male	16 992	44 %
*Current situation*		
Caregiving (home)	733	1·9 %
Retired	904	2·3 %
Unable to work	8586	22 %
Unemployed	1466	3·8 %
Worker	26 938	70 %
*Marital status*		
Divorced\separated\widowed	8074	21 %
Married	25 801	67 %
Single	4752	12 %
*Level of education*		
Elementary	441	1·1 %
High school	7425	19 %
Technical school	8564	22 %
College	3664	9·5 %
University certificate	3559	9·2 %
Bachelor’s degree	9392	24 %
Graduate studies	5582	14 %
*Age at initial data collection (years)*		
40–44	4457	12 %
45–49	8595	22 %
50–54	9352	24 %
55–59	6505	17 %
60–64	5395	14 %
65+	4323	11 %
*Household yearly income (Canadian dollars)*		
Less than 25 000	2767	7·2 %
25 000–49 999	4926	13 %
50 000–74 999	8072	21 %
75 000–99 999	8399	22 %
100 000–149 999	6726	17 %
More than 150 000	7737	20 %
*Number of dependents in the household*		
1	8393	22 %
2	15 684	41 %
3	6082	16 %
4	5653	15 %
5+	2815	7·3 %
*All*	38 627	100 %

**Table 3:** Demographic and socio-economic characteristics distribution of participants by food environment trajectories

Characteristic	Overall, *N* 386 271		Limited access throughout (*n* 7771) (20 %)		Limited access improving (*n* 8728) (23 %)		Good access diminishing (*n* 10 628) (28 %)		Good access throughout (*n* 10 833) (28 %)		Low access (*n* 667) (1·7 %)	
	***n***	%	***n***	%	***n***	%	***n***	%	***n***	%	***n***	%
*Sex at birth*												
Female	21 635	56 %	4168	54 %	4863	56 %	6079	57 %	6128	57 %	397	60 %
Male	16 992	44 %	3603	46 %	3865	44 %	4549	43 %	4705	43 %	270	40 %
*Age at initial data collection (years)*												
40–44	4457	12 %	941	12 %	1033	12 %	1163	11 %	1221	11 %	99	15 %
45–49	8595	22 %	1890	24 %	2063	24 %	2311	22 %	2156	20 %	175	26 %
50–54	9352	24 %	2043	26 %	2034	23 %	2503	24 %	2614	24 %	158	24 %
55–59	6505	17 %	1130	15 %	1488	17 %	1877	18 %	1920	18 %	90	13 %
60–64	5395	14 %	949	12 %	1183	14 %	1552	15 %	1622	15 %	89	13 %
65+	4323	11 %	818	11 %	927	11 %	1222	11 %	1300	12 %	56	8·4 %
*Level of education*												
Elementary	441	1·1 %	73	0·9 %	79	0·9 %	144	1·4 %	132	1·2 %	13	1·9 %
High school	7425	19 %	1675	22 %	1755	20 %	1912	18 %	1886	17 %	197	30 %
Technical school	8564	22 %	1815	23 %	2036	23 %	2373	22 %	2146	20 %	194	29 %
College	3664	9·5 %	817	11 %	841	9·6 %	979	9·2 %	977	9·0 %	50	7·5 %
University certificate	3559	9·2 %	679	8·7 %	820	9·4 %	1040	9·8 %	954	8·8 %	66	9·9 %
Bachelor’s degree	9392	24 %	1849	24 %	2011	23 %	2584	24 %	2840	26 %	108	16 %
Graduate studies	5582	14 %	863	11 %	1186	14 %	1596	15 %	1898	18 %	39	5·8 %
*Marital status*												
Divorced\separated\widowed	8074	21 %	1456	19 %	1874	21 %	2300	22 %	2299	21 %	145	22 %
Married	25 801	67 %	5748	74 %	6034	69 %	7028	66 %	6553	60 %	438	66 %
Single	4752	12 %	567	7·3 %	820	9·4 %	1300	12 %	1981	18 %	84	13 %
*Current situation*												
Caregiving (home)	733	1·9 %	184	2·4 %	148	1·7 %	200	1·9 %	180	1·7 %	21	3·1 %
Retired	904	2·3 %	142	1·8 %	162	1·9 %	270	2·5 %	312	2·9 %	18	2·7 %
Unable to work	8586	22 %	1632	21 %	1950	22 %	2440	23 %	2418	22 %	146	22 %
Unemployed	1466	3·8 %	214	2·8 %	268	3·1 %	436	4·1 %	513	4·7 %	35	5·2 %
Working	26 938	70 %	5599	72 %	6200	71 %	7282	69 %	7410	68 %	447	67 %
*House yearly income (Canadian dollars)*												
Less than 25 000	2767	7·2 %	314	4·0 %	458	5·2 %	749	7·0 %	1165	11 %	81	12 %
25 000–49 999	4926	13 %	1121	14 %	1137	13 %	1377	13 %	1244	11 %	47	7·0 %
50 000–74 999	8072	21 %	1369	18 %	1784	20 %	2294	22 %	2464	23 %	161	24 %
75 000–99 999	8399	22 %	1678	22 %	1860	21 %	2256	21 %	2438	23 %	167	25 %
100 000–149 999	6726	17 %	1471	19 %	1623	19 %	1841	17 %	1687	16 %	104	16 %
More than 150 000	7737	20 %	1818	23 %	1866	21 %	2111	20 %	1835	17 %	107	16 %
*Number of dependents in the household*												
1	8393	22 %	1179	15 %	1667	19 %	2409	23 %	2986	28 %	152	23 %
2	15 684	41 %	3148	41 %	3682	42 %	4357	41 %	4249	39 %	248	37 %
3	6082	16 %	1362	18 %	1346	15 %	1712	16 %	1560	14 %	102	15 %
4	5653	15 %	1386	18 %	1353	16 %	1436	14 %	1390	13 %	88	13 %
5+	2815	7·3 %	696	9·0 %	680	7·8 %	714	6·7 %	648	6·0 %	77	12 %
*All*	100 %		100 %		100 %		100 %		100 %		100 %	

### Description of the food environment trajectories

**Fig. 2:**
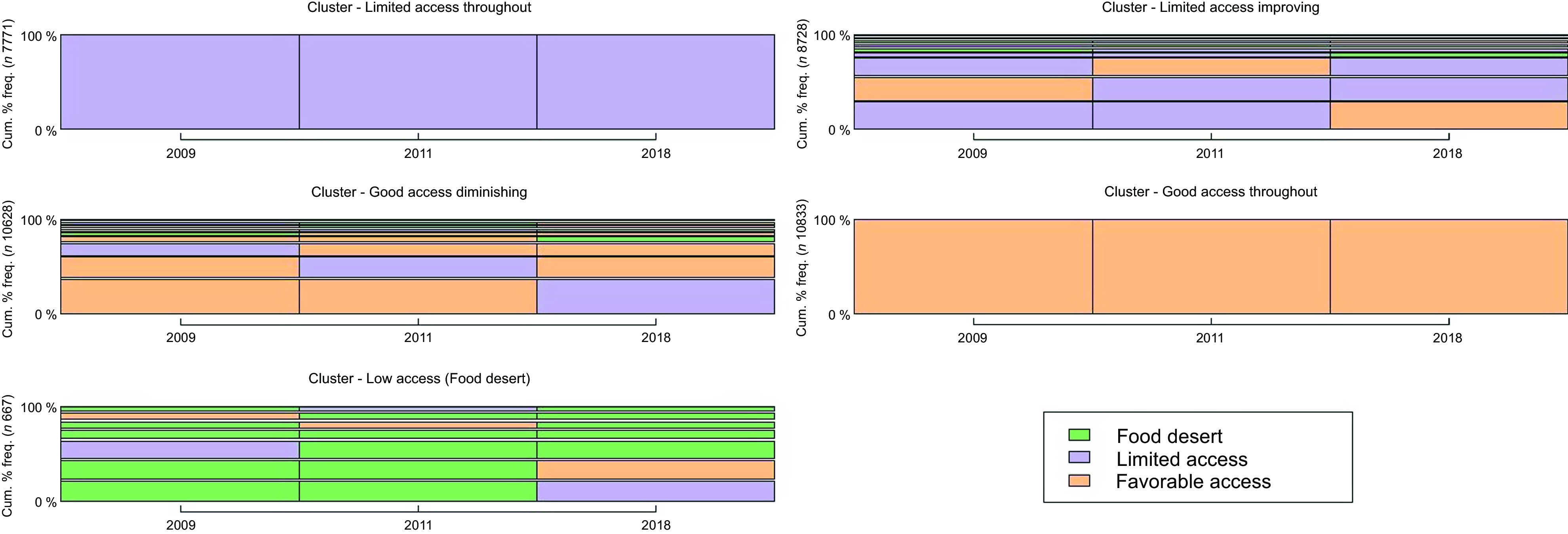
Food environment trajectory typologies. This figure is used for a better visualisation of the ten typical sequences of each class. In other words, the ten most frequent sequences in each class that are representative of the class

Results from the sequence class analysis revealed five types of access to food stores (Fig. 2). All participants fell into one of these five trajectories of access to food stores. Each food environment trajectory has its characteristics which are presented below.

**Fig. 3:**
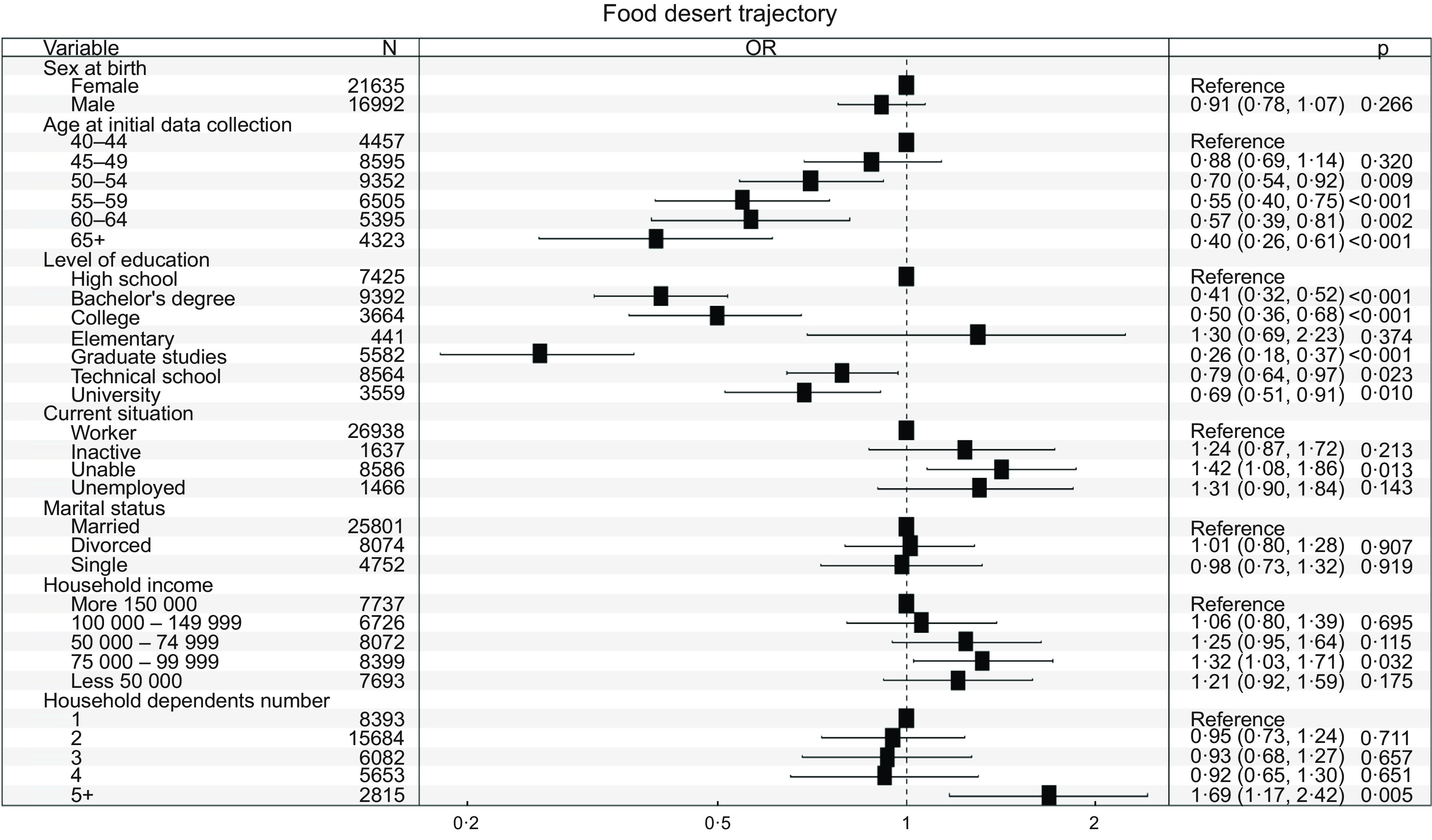
Factors of exposure within trajectories characterised by food desert environments

**Trajectory 1 – Limited access throughout:** This food environment trajectory includes participants who experienced a stable food environment trajectory between 2009 and 2018 characterised by low access to food stores throughout the studied period. Nearly, all these participants in this trajectory lived in food environments with low access to food stores even if/when they moved.

**Trajectory 2 – Limited access improving:** Trajectory 2 includes participants who had low access to food stores initially, but for some participants, food access improved over time. Between 2011 and 2018, food store access of individuals in this trajectory oscillated between low access and favourable access. In 2009, nearly 65 % of people in this trajectory lived in areas with low access to food stores, while 5 % were in food deserts and 30 % were in areas with favourable access to food stores. In 2018, at the end of the observation period, 58 % lived in areas with low access to food stores, 6 % lived in an area considered a food desert and 36 % had favourable access to food stores.

**Trajectory 3 – Good access diminishing:** This food environment trajectory encompasses participants who initially experienced favourable access to food stores, only to witness a subsequent deterioration. Specifically, this category includes individuals residing in areas where access to food stores was initially favourable but underwent a decline after 2011 (Fig. 2). At the beginning of the observation period in 2009, 80 % of participants in this trajectory resided in DA with favourable access to food stores, reaching 100 % in 2011. However, by the conclusion of the observation period in 2018, nearly 60 % found themselves in areas characterised by low access to food stores.

**Trajectory 4 – Good access throughout:** This food environment trajectory is characterised by favourable access to food stores throughout the study period. It includes participants who experienced a stable food environment trajectory between 2009 and 2018, with nearly all of them living in environments with favourable access to food stores.

**Trajectory 5 – Low access (food desert):** Participants in this food environment trajectory remained in a food desert throughout the study period. This trajectory includes participants that had largely stable food environment trajectories between the three types of food environments. By 2011, almost all the participants that started in a food desert had better access to food stores in this trajectory. Conditions improved both for those who were in food deserts and those who started with low access to food stores (Table 2). This class includes a high proportion of women and people from households that earned less than 100 000 CAD$ per year (Table 3).

### Demographic and socio-economic factors associated with the disadvantaged food environment trajectory

Out of the five food environment trajectories, two can be classified as disadvantaged. These are the food environment trajectory with low access to food stores (trajectory 5) and the food environment trajectory with limited access to food stores (trajectory 1). The only difference between the participants in these two trajectories was based on the material deprivation index. The participants in both trajectories lived in areas with low food access, whereas trajectory 5 participants were also living in materially deprived environments. To analyse the determinants of belonging to the disadvantaged environment trajectory, we created a trajectory that includes participants from the low access to food stores (food desert) group compared with the rest of the sample.

#### Disadvantaged food environment trajectory

Participants’ sex and marital status were not found to be associated with experiencing a disadvantaged food environment trajectory (Fig. 3). However, individuals who were divorced had slightly higher odds (OR = 1·01, adjusted CI = 0·80–1·97) of experiencing a disadvantaged food environment trajectory compared with those who were married. Participants who were 60 years of age and above had lower odds of experiencing a disadvantaged food environment trajectory when compared with those who were aged 40–44 years. The OR was 0·40 with a CI of 0·26–0·61. This suggests that increased age is associated with a decreased likelihood of having a disadvantaged food environment trajectory. The association between the current employment situation and the disadvantaged food environment trajectory was weak. The study found that unemployed participants (OR = 1·31 CI = 0·90–1·84) or participants who were unable to work (OR = 1·42 CI = 1·08–1·86) were more likely to experience a disadvantaged food environment trajectory than those who were employed. Inactive participants (OR = 1·24 CI = 0·80–1·72) were also more likely to experience a disadvantaged food environment trajectory compared with those who were employed.

There is a significant association between the disadvantaged food environment trajectory and the level of education, annual household income, and number of dependents in the household (*P* < 0·001). Having an annual household income of CAD 100 000 or more was found to decrease the likelihood of experiencing a disadvantaged food environment trajectory. For instance, individuals from households earning less than CAD 100 000 per year (OR = 1·42 CI = 1·08–1·86) had higher chances of experiencing a disadvantaged food environment trajectory compared with those from households earning more than CAD 150 000 per year. Additionally, the odds of experiencing a disadvantaged food environment trajectory increased when there were more than five persons in the household. Households with five or more people (OR = 1·69 CI = 1·17–2·46) had higher odds of experiencing a disadvantaged food environment trajectory compared with households with only one person. The level of education a person attains is related to their access to healthy food options. People with only elementary education had a higher odd (OR = 1·30 CI = 0·69–2·23) to experience a disadvantaged food environment trajectory compared with those with a high school education. However, those with a college-level education, graduate degrees or university certificates had a lower odd to experience a disadvantaged food environment trajectory compared with those with only a high school education.

## Discussion

This study aimed to create a socio-economic-based typology of longitudinal exposure to the food environment from 2009 to 2018. We used sequence analysis to create five different food environment trajectories with varying levels of food access over a period of 9 years. While some participants had constant access to food stores, others experienced fluctuations between 2009 and 2018. We found that age, employment status, education level, number of dependents in the household, and household income and age of participants were the most significant determinants of a disadvantaged food environment trajectory.

### Demographic factors related to the disadvantaged food environment trajectory

Between 2009 and 2018, the percentage of people living in food deserts with limited access to food increased from 43·45 % to 50·62 %, according to the CARTaGENE population-based cohort. This trend is also evident in many North American and European countries^(17,26,31,44)^. It is important to investigate the deterioration of food environments to better understand it and guide decision-makers in developing strong public policies to ensure food access for everyone. In this study, the use of sequence analysis allowed us to create longitudinal food environment indicators, which helped us understand the different trajectories of food store access in Quebec over time.

Demographic factors have been very rarely used to explain disparities in unhealthy food environment trajectories or food environment longitudinal indicators, making it difficult to compare our results with other studies. The CARTaGENE data provide us with this opportunity to assess the odds that participants have of belonging to the five types of food environment trajectories we created, based on their demographic characteristics rather than on community characteristics.

Our findings indicate that women in our cohort are more likely to reside in an unfavourable food environment trajectory. This implies that, through their residential trajectories, women have greater exposure to areas with limited access to food stores compared with men. However, this disparity is not statistically significant. A physical environment study conducted on the Multi-Ethnic Study of Atherosclerosis (MESA) cohort did not observe any discrepancy in access to healthy food environments between sexes, as seen through descriptive analyses^(45)^. Nevertheless, another study of the same cohort reports a weak correlation between the participants’ sex and the local food environment^(46)^. Our study found that age was the most significant factor associated with a disadvantaged food environment trajectory. As young people grew older, they were more likely to experience this trajectory. In fact, from the age of 40 years onwards, the likelihood of experiencing a disadvantaged food environment trajectory increased significantly. These findings are contrary to those reported in the 40-year food environment study of the Framingham Heart Study cohort. Researchers did not find a consistent relation between the sex and age of participants and access to a supermarket or fast-food outlets^(26)^.Finally, we found that married individuals generally had better access to healthy food options when compared with those who were divorced, separated or widowed. This trend was observed to be consistent with the number of dependents in the household, as households with more than five dependents had a higher probability of experiencing a disadvantaged food environment.

### Socio-economic factors are related to the disadvantaged food environment trajectory

There have been several studies that indicate significant differences in the trajectories of unhealthy food environments between disadvantaged and affluent socio-economic communities^(17,26,28,31)^. A previous study conducted in Australia revealed that irrespective of the area’s level of food access or dietary status, the food supply in poorer communities was less health-promoting as compared with that of their affluent counterparts in the long run^(28)^. Our results further highlight the socio-economic inequalities based on the annual household income and the current employment status of participants. Participants who were unemployed, inactive or unable had higher chances of experiencing a disadvantaged food environment trajectory as compared with those who were employed. Furthermore, individuals belonging to households earning less than 100 000 $CAD annually were found to have a greater likelihood of experiencing a disadvantaged food environment trajectory as compared with those in higher-income households. It has been established that socio-economic disadvantage is linked with an unhealthy food environment over a long period^(17,34,35,47)^. Additionally, other studies have found that low median household income is associated with a higher concentration of fast-food outlets in the neighbourhood over a long period^(12,33,47)^.

The results of our study indicate that the level of education of participants is linked to socio-economic inequality and has an impact on the food environment trajectories. While a few studies have analysed individual socio-economic characteristics of food environment trajectories, most studies focus on median household income per DA. Two American studies found that individuals with low education levels are more likely to experience a persistent low-access trajectory to supermarkets. Our study also suggests a strong relationship between the level of education and disadvantaged food trajectory. However, the direction of this relationship is not entirely clear. On the one hand, participants with a high school level education are more exposed to a disadvantaged food environment trajectory than those with elementary education levels. On the other hand, participants with college-level education are more exposed to a disadvantaged food environment trajectory than participants with technical education levels. Finally, some high-income residential neighbourhoods also lack access to food stores^(38)^. However, one study on obesogenic environments found that disadvantaged communities have fewer supermarkets than advantaged communities^(48)^.

This study has limitations due to its methodology, dataset and food environment indicators. The study relied on data from the CARTaGENE cohort for only three time points: 2009, 2011 and 2018. This limited data availability between 2009 and 2018 reduced the accuracy of the food environment trajectories. Additionally, the food environment indicators used in this study only accounted for categories of food stores like grocery stores, supermarkets, farmer’s markets, and fruit and vegetable shops^(49)^. These categories were characterised as contributing healthy foods to the food environment. However, research shows that supermarkets and grocery stores also offer a variety of unhealthy, highly processed foods^(26)^. Therefore, adding in-store indicators to the physical access indicators would improve the food indicators and the food environment trajectories.

Another limitation is that the CARTaGENE cohort was designed to recruit adults between 40 and 69 years from metropolitan areas in the province of Quebec. Therefore, the results cannot be generalised to the general population. While this study has its limitations, it boasts several notable strengths. The methods employed to create food environment trajectories were reliable, and a large sample size was included. The observed socio-economic disparities in food environments may help to shed light on the varying rates of obesity among different socio-economic groups. The food environment trajectories established in this study hold promise for analysing chronic diseases in CARTaGENE cohort participants. Ongoing research will explore the potential correlation between these trajectories and the weight status of participants in the CARTaGENE cohort.

### Conclusions

The literature on food environments generally focuses on socio-economic disparities between affluent and disadvantaged communities when it comes to access to healthy food stores. However, our results lead to further analyses of the trajectories of individuals’ food environments, which reveal socio-economic inequalities related to individual demographic and socio-economic characteristics. Age, level of education, current employment situation, annual household income and the number of dependents in the household appear to affect the food environment trajectories among the CARTaGENE cohort.

This is one of the few studies that create longitudinal food environment indicators, identify food environment trajectories, and their individual demographic and socio-economic determinants. This study shows that socio-economic disadvantage was associated with a disadvantaged food environment trajectory. Thus, the promotion of healthy food environments requires both the zoning of food outlets in territorial planning and the reduction of socio-economic inequalities. Our results provide insights into the promotion of healthy eating environments in Québec and help to better identify the disadvantaged groups that are most exposed to unhealthy food environments.

Public policies should aim to improve food environments, especially in neighbourhoods with vulnerable communities.

## Data Availability

Restrictions apply to the availability of these data. Data were obtained from CARTaGENE, CHU Ste-Justine, and data requests should be directed to them (https://www.cartagene.qc.ca, accessed on 4 January 2022).
